# Effect of anterior chamber depth on the choice of intraocular lens calculation formula

**DOI:** 10.1371/journal.pone.0189868

**Published:** 2017-12-18

**Authors:** Soonwon Yang, Woong-Joo Whang, Choun-Ki Joo

**Affiliations:** 1 Department of Ophthalmology and Visual Science, Seoul St. Mary’s Hospital, College of Medicine, The Catholic University of Korea, Seoul, Republic of Korea; 2 Department of Ophthalmology and Catholic Institute for Visual Science, College of Medicine, The Catholic University of Korea, Seoul, Republic of Korea; Washington University in Saint Louis, UNITED STATES

## Abstract

**Purpose:**

To investigate the effect of anterior chamber depth (ACD) on the refractive outcomes of the SRK/T, Holladay 1, Hoffer Q and Haigis formulae in short, normal, long and extremely long eyes.

**Methods:**

This retrospective study involved patients who had uncomplicated cataract surgery. Preoperative axial length (AL) was divided into four subgroups: short (< 22.00 mm), normal (22.00–24.49 mm), long (24.50–25.99 mm), extremely long (≥ 26.00 mm). Preoperative ACD was divided into three subgroups: < 2.5, 2.50–3.49, and ≥ 3.5 mm. Median absolute errors (MedAEs) predicted by the SRK/T, Holladay 1, Hoffer Q and Haigis formulae were compared with the Friedman test. Post-hoc analysis involved the Wilcoxon signed rank test with a Bonferroni adjustment. Correlations between ACD and the predictive refractive errors of the four formulas were analyzed.

**Results:**

In short eyes with an ACD < 2.5 mm, the Haigis formula revealed the highest MedAE. The difference in MedAE with the Hoffer Q formula (which had the lowest MedAE) was statistically significant (P = 0.002). In normal eyes, the Haigis formula significantly differed from the Holladay 1 (P = 0.002) and Hoffer Q (P = 0.005) formulae in the ACD < 2.5 mm group. In long eyes and extremely long eyes with an ACD ≥ 3.5 mm, the differences in MedAEs were statistically significant (P = 0.018, P = 0.001, respectively) and the Haigis formula had the lowest MedAEs in both subgroups (0.29 D, 0.30 D, respectively). In the total of 1,123 eyes, refractive errors predicted by the Haigis formula showed a significant negative correlation with the ACD (R^2^ = 0.002, P = 0.047).

**Conclusions:**

The Hoffer Q formula is preferred over other formulae in short eyes with an ACD shallower than 2.5 mm. In short and normal eyes with an ACD < 2.5 mm the Haigis formula might underestimate ELP. The Haigis formula is the preferred choice in eyes with an AL ≥ 24.5 mm and an ACD ≥ 3.5 mm.

## Introduction

With modern surgical techniques, patients have increasingly higher refractive expectations. To achieve optimal refractive outcomes, accurate intraocular lens (IOL) power calculation is important.[1,2] IOL power is calculated using preoperative biometric measurements such as axial length (AL), corneal power (K), anterior chamber depth (ACD) and an estimation of postoperative effective lens position (ELP).[3,4] Previous studies have reported that every 1.0 mm erroneous measurements of corneal radius, AL and ACD can result in 5.7 D, 2.7D, and 1.5 D of refractive error, respectively.[5] Precise AL measurements are possible using the IOLMaster (Carl Zeiss Meditec, Jena, Germany).[6,7] Thus, ACD contributes to residual refractive error a lot more than AL. Olsen showed that contribution to error from ACD, AL, and corneal power is 42, 36, and 22%, respectively.[5]

Modern IOL calculation formulae show similarly accurate refractive outcomes in eyes with a normal range of AL.[4,8] However, the accuracy of these formulae differ in eyes with short and long AL.[4,8–10] The Hoffer Q formula is most accurate in eyes with a short AL[3,4,11] and the SRK/T and Haigis formulae are best for eyes with a long AL.[3,12–15] Based on these studies, Eom et al. showed that the differences between the predicted refractive errors of the Hoffer Q and Haigis formulae increased as ACD decreased in short eyes[16], and Miraftab et al. reported that the Haigis formula is the preferred choice in patients with a normal AL and ACD exceeding 3.5 mm.[17] However, no other studies have directly compared the refractive outcomes of modern IOL calculation formulae according to the ACD in short, normal and long eyes.

The Hoffer Q, SRK/T and Holladay 1 are third-generation formulae that rely on AL and corneal height for the estimation of postoperative ELP. The Haigis formula is a fourth-generation formula that considers AL and preoperative ACD to predict ELP.[5] The accuracy of these formulae may differ according to the ACD even in eyes with the same AL and K. This study compared the accuracy of the SRK/T, Holladay 1, Hoffer Q and Haigis formulae, and evaluated the effect of ACD on the refractive outcomes in short, normal, long and extremely long eyes.

## Materials and methods

A retrospective chart review involved patients who underwent uncomplicated cataract surgery with the EC-1PAL lens (Aaren Scientific Inc., Ontario, CA, USA) implantation at our center from January 1, 2013 to December 31, 2015. Surgeries were performed by one surgeon (CKJ). All eyes underwent sutureless 2.2 mm micro coaxial cataract surgery under topical anesthesia. Inclusion criteria were an availability of preoperative AL (at least three valid measurements with a signal-to-noise ratio (SNR) above 1.5 for a single measurement and a signal-to-noise ratio > 2.0 for the composite signal), corneal power, preoperative ACD data and 3-month postoperative refraction data. Exclusion criteria were patients with best-corrected visual acuities (BCVA) < 20/40 after cataract surgery, previous ocular surgery (e.g., refractive surgery) and postoperative complications. Institutional Review Board approval was from the Catholic University of Korea Seoul St. Mary’s Hospital, Seoul, Republic of Korea. All research and data collection followed the tenets of the Declaration of Helsinki. AL, corneal power (K), and ACD were measured with IOL Master version 5.4 (Zeiss AG, Jena, Germany). Optimized IOL constants were used for each formula. The a0, a1 and a2 constants were 1.22, 0.40 and 0.10, respectively, for the Haigis formula and the pseudophakic ACD (pACD) was 5.37 for the Hoffer Q formula. In addition, the A-constant for SRK/T was 118.7 and sf = 1.63 for the Holladay 1 formula.[18] For analysis, AL was divided into four subgroups: short (< 22.0 mm), normal (22.0 ≤ AL < 24.5 mm), long (24.5 ≤ AL < 26.0 mm), extremely long (≥ 26.0 mm) and preoperative anterior chamber depth (ACD) was divided into three subgroups: < 2.5, 2.50–3.49, and ≥ 3.5 mm.

The main outcome measures were the median absolute error (MedAE) calculated as the absolute median deviation from the predicted postoperative refractive outcome, the mean absolute error (MAE) calculated as the absolute mean deviation from the predicted postoperative refractive outcome and the refractive error (RE) calculated as the arithmetic mean deviation from the predicted postoperative refractive outcome. To compare errors with these formulas, we used the Friedman non-parametric test of the MedAE. Post hoc analysis was used for multiple comparisons among formulae. We used the Wilcoxon signed rank test with the Bonferroni adjustment for post-hoc analysis to compare the MedAE of each formula in the different ACD subgroups. The Pearson correlation coefficient determined the correlation of ACD and the RE. IBM/SPSS software version 21 (IBM/SPSS Inc. Chicago, IL, USA) was used to perform the statistical analyses. In all analyses, the level of significance was set at 0.05.

## Results

Data from 1,123 eyes were extracted for analysis based on the inclusion criteria. Nearly half (48.9%) of the samples were right eyes. The majority (63.1%) of the patients were female. The mean patient age was 67.11 ± 11.1 years (range, 40–94 years). The mean corneal power was 44.26 ± 1.51 diopter (D; range, 41.25–49.97 D), the mean ACD was 3.18 ± 0.42 mm (range, 2.02–4.42 mm), and the mean axial length was 23.93 ± 1.46 mm (range, 20.59–30.60 mm).

**Fig 1:**
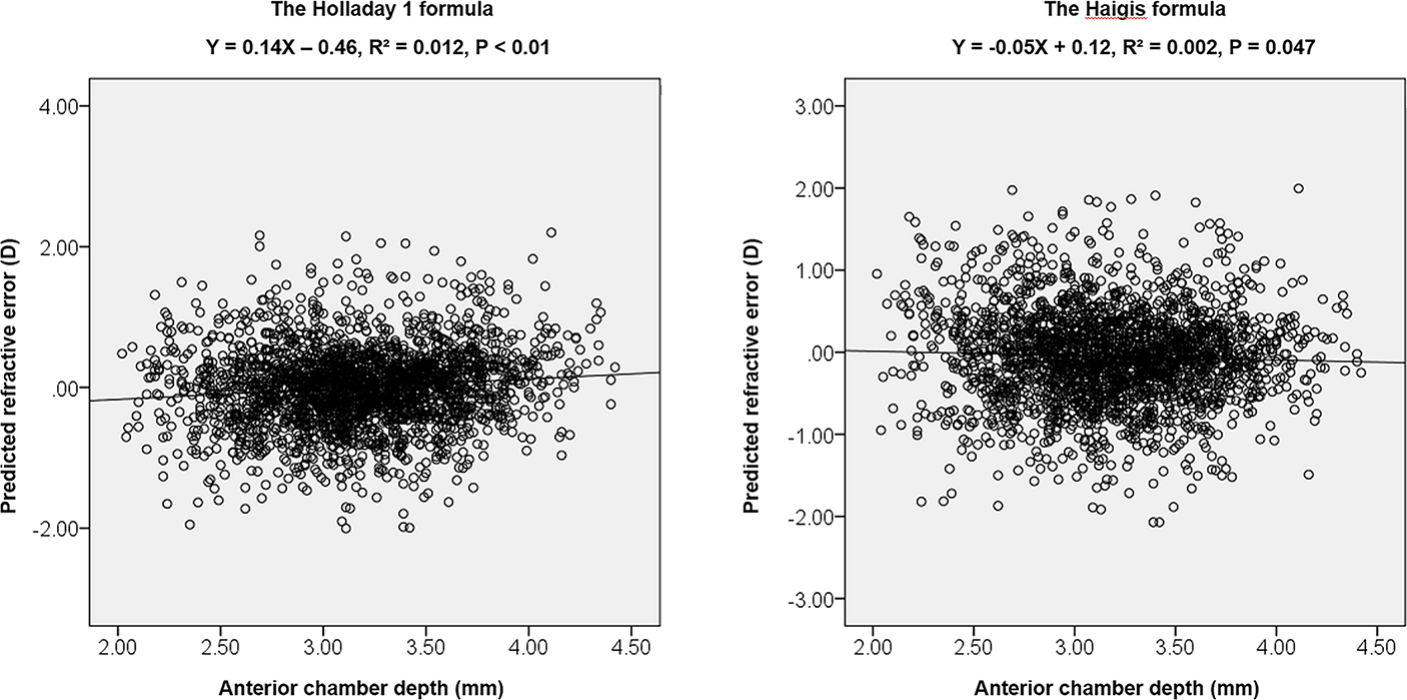
Linear correlation between the anterior chamber depth and predicted refractive errors by the Holladay 1 and Haigis formulae.

Table 1 indicates the MedAEs, MAEs and mean predicted refractive errors of subgroups according to ACD values (2.5 mm and 3.5 mm) determined by the four formulas in total 1,123 eyes. The mean predicted refractive errors for each formula were zeroed out. There were statistically significant differences in MedAEs in the four formulas in all three ACD subgroups (P = 0.001, P = 0.005, P = 0.013, respectively). Post hoc analysis with Wilcoxon signed-rank test was conducted with a Bonferroni correction applied, resulting in a significance level set at P < 0.0083. In the ACD < 2.5 mm subgroup, the Haigis formula had the highest MedAE and the differences in MedAEs with the other three formulas were statistically significant (P = 0.006, P<0.001 and P<0.001, respectively). In the ACD 2.50–3.49 mm subgroup, there were significant differences in MedAEs between the Holladay 1 formula and the other three formulas (P<0.001). Lastly, the Haigis formula had the lowest MedAE in the ACD ≥ 3.5 mm subgroup and the differences of MedAEs with the other three formulas were statistically significant (P<0.0083). Refractive errors predicted by the Haigis formula showed a significant negative correlation with the ACD (R^2^ = 0.002, P = 0.047; Fig 1), while the Holladay 1 formula showed a significant positive correlation with the ACD (R^2^ = 0.012, P<0.01; Fig 1). In the Haigis formula, the refractive outcome was hyperopic when the ACD was shallower than 2.40 mm and myopic when the ACD was deeper than 2.40 mm. In contrast, the refractive outcome was myopic when the ACD was shallower than 3.29 mm and hyperopic when the ACD was deeper than 3.29 mm in the Holladay 1 formula. The refractive errors predicted by the SRK/T and Hoffer Q formula showed no correlation with the ACD.

**Table 1:** Comparison of the median bbsolute error, mean absolute error and predicted Refractive errors of subgroups based on the anterior chamber depth among the four formulas for intraocular lens power calculation in 1,123 eyes (Friedman test).

	Mean±SD				
	SRK/T	Holladay 1	Hoffer Q	Haigis	P-value
**ACD < 2.5 mm**					
**(n = 79)**					
**MedAE, D**	0.38	0.31	0.35	0.46	0.001
**MAE±SD, D**	0.49±0.43	0.44±0.39	0.48±0.42	0.55±0.41	
**RE±SD, D (range)**	0.06±0.64	0.03±0.59	-0.07±0.64	0.21±0.64	
	(-1.42~1.28)	(-1.73~1.12)	(-2.05~1.00)	(-1.82~1.31)	
±0.25 D (%)	36.7	41.8	41.8	39.2	
±0.50 D (%)	59.5	62.0	63.3	60.8	
±1.00 D (%)	89.9	94.9	94.9	92.4	
>±2.00 D (%)	0	0	0	0	
**2.5 mm ≤ ACD < 3.5 mm**					
**(n = 808)**					
**MedAE, D**	0.30	0.29	0.33	0.32	0.005
**MAE±SD, D**	0.39±0.34	0.38±0.35	0.39±0.37	0.39±0.33	
**RE±SD, D (range)**	-0.01±0.50	-0.04±0.52	-0.02±0.51	-0.02±0.51	
	(-1.97~2.03)	(-1.87~1.93)	(-1.98~1.73)	(-1.87~1.43)	
±0.25 D (%)	42.9	43.2	42.5	41.1	
±0.50 D (%)	70.5	71.8	70.0	69.7	
±1.00 D (%)	94.7	94.8	94.2	94.8	
>±2.00 D (%)	0	0	0	0	
**3.5 mm ≤ ACD**					
**(n = 236)**					
**MedAE, D**	0.27	0.28	0.31	0.23	0.013
**MAE±SD, D**	0.37±0.34	0.36±0.29	0.39±0.39	0.34±0.32	
**RE±SD, D (range)**	-0.01±0.50	0.12±0.44	0.09±0.48	-0.01 ±0.47	
	(-1.98~2.03)	(-1.58~2.12)	(-2.05~1.98)	(-2.12~1.99)	
±0.25 D (%)	41.1	41.1	37.7	47.5	
±0.50 D (%)	70.3	70.8	67.4	75.4	
±1.00 D (%)	92.8	92.4	90.7	94.5	
>±2.00 D (%)	0.004	0.004	0.008	0.004	

SD = standard deviation, ACD = anterior chamber depth, MedAE = median absolute error, MAE = mean absolute error, RE = mean predicted refractive error, D = diopters

Table 2 shows the MedAEs, MAEs and mean predicted refractive errors of subgroups according to ACD values (2.50 mm and 3.50 mm) determined by the four formulas in eyes with an AL < 22.0 mm. There was a statistically significant difference in MedAE in four formulas in ACD < 2.5 mm (P = 0.004). Post hoc analysis revealed that the Haigis formula had the highest MedAE and the difference in MedAE with the Hoffer Q formula (which had the least MedAE) was statistically significant (P = 0.002). Linear regression analysis showed no correlation between the ACD and the refractive errors predicted by the four formulas in this subgroup.

**Table 2:** Comparison of the median absolute error, mean absolute error and predicted refractive errors of subgroups based on the anterior chamber depth among the four formulas for intraocular lens power calculation in eyes with axial length < 22.0 mm (Friedman test).

	Mean±SD				
	SRK/T	Holladay 1	Hoffer Q	Haigis	P-value
**ACD < 2.5 mm**					
**(n = 32)**					
**MedAE, D**	0.47	0.48	0.30	0.63	0.004
**MAE±SD, D**	0.58±0.51	0.55±0.45	0.51±0.53	0.65±0.43	
**RE±SD, D (range)**	0.12±0.55	0.03±0.52	-0.11±0.54	0.16±0.62	
	(-1.42~0.58)	(-1.73~0.54)	(-2.05~0.35)	(-1.82~0.75)	
±0.25 D (%)	37.5	43.8	46.9	46.9	
±0.50 D (%)	56.3	53.1	59.4	62.5	
±1.00 D (%)	84.3	90.6	93.8	93.8	
>±2.00 D (%)	0	0	0	0	
**2.5 mm ≤ ACD < 3.5 mm**					
**(n = 58)**					
**MedAE, D**	0.41	0.39	0.30	0.37	0.316
**MAE±SD, D**	0.53±0.43	0.48±0.38	0.46±0.38	0.49±0.38	
**RE±SD, D (range)**	0.04±0.63	-0.02±0.62	-0.15±0.63	-0.12±0.60	
	(-1.69~1.36)	(-1.80~1.17)	(-1.90~1.01)	(-1.87~1.08)	
±0.25 D (%)	39.7	39.7	41.3	39.7	
±0.50 D (%)	60.3	62.1	62.1	62.1	
±1.00 D (%)	88.2	91.3	94.8	93.1	
>±2.00 D (%)	0	0	0	0	

SD = standard deviation, ACD = anterior chamber depth, MedAE = median absolute error, MAE = mean absolute error, RE = mean predicted refractive error, D = diopters

The MedAEs, MAEs and mean predicted refractive errors of subgroups according to ACD values (2.50 mm and 3.50 mm) determined by the four formulas in eyes with an AL 22.00–24.49 mm are summarized in Table 3. In the ACD < 2.5 mm and ACD 2.50–3.49 mm subgroups, there were statistically significant differences in MedAEs in four formulas (P = 0.012 and P = 0.001, respectively). In the ACD < 2.5 mm subgroup, the Haigis formula had the highest MedAE. Post hoc analysis showed that the MedAE with the Haigis formula differed significantly from the Holladay 1 (P = 0.002) and Hoffer Q (P = 0.005) formulas. In the ACD 2.50–3.49 mm subgroup, MedAEs and MAEs in the four formulas were comparable. The Holladay 1 formula had the least MedAE which differed significantly from the other three formulas (SRK/T; P = 0.003, Hoffer Q; P = 0.001, Haigis; P = 0.001, respectively). Linear regression analysis revealed a significant correlation between the ACD and the refractive errors predicted by the Holladay 1 and Haigis formulas. Refractive errors predicted by the Holladay 1 formula had a significant positive correlation with the ACD (R^2^ = 0.003, P = 0.015), while refractive errors predicted by the Haigis formula had a significant negative correlation with the ACD (R^2^ = 0.009, P<0.001).

**Table 3:** Comparison of the median absolute error, mean absolute error and predicted refractive errors of subgroups based on the anterior chamber depth among the four formulas for intraocular lens power calculation in eyes with an axial length 22.00–24.49 mm (Friedman test).

	Mean±SD				
	SRK/T	Holladay 1	Hoffer Q	Haigis	P-value
**ACD < 2.5 mm**					
**(n = 47)**					
**MedAE, D**	0.31	0.29	0.34	0.42	0.012
**MAE±SD, D**	0.43±0.38	0.43±0.39	0.49±0.39	0.51±0.40	
**RE±SD, D (range)**	0.02±0.63	0.03±0.59	-0.04±0.59	0.24±0.60	
	(-0.98~1.28)	(-1.02~1.12)	(-1.00~1.00)	(-0.96~1.31)	
±0.25 D (%)	36.2	40.4	38.3	34.1	
±0.50 D (%)	61.7	68.1	66.0	60.0	
±1.00 D (%)	93.6	97.8	95.7	91.5	
>±2.00 D (%)	0	0	0	0	
**2.5 mm ≤ ACD < 3.5 mm**					
**(n = 610)**					
**MedAE, D**	0.30	0.28	0.33	0.32	0.001
**MAE±SD, D**	0.38±0.33	0.37±0.33	0.39±0.29	0.39±0.34	
**RE±SD, D (range)**	-0.01±0.55	-0.04±0.54	-0.05±0.54	-0.02±0.56	
	(-0.98~1.26)	(-1.06~1.33)	(-1.23~1.01)	(-1.29~1.26)	
±0.25 D (%)	44.8	45.1	44.6	41.6	
±0.50 D (%)	73.8	74.6	73.8	72.0	
±1.00 D (%)	96.7	96.3	95.9	95.5	
>±2.00 D (%)	0	0	0	0	
**3.5 mm ≤ ACD**					
**(n = 85)**					
**MedAE, D**	0.25	0.25	0.29	0.22	0.252
**MAE±SD, D**	0.38±0.40	0.34±0.27	0.40±0.39	0.32±0.28	
**RE±SD, D (range)**	-0.01±0.55	0.17±0.39	-0.04±0.48	-0.02±0.43	
	(-1.22~1.28)	(-1.17~1.33)	(-1.23~1.40)	(-1.44~1.38)	
±0.25 D (%)	44.7	47.1	45.9	48.2	
±0.50 D (%)	77.6	75.3	77.6	80.0	
±1.00 D (%)	97.6	95.3	95.3	94.1	
>±2.00 D (%)	0	0	0	0	

SD = standard deviation, ACD = anterior chamber depth, MedAE = median absolute error, MAE = mean absolute error, RE = mean predicted refractive error, D = diopters

In eyes between 24.5 mm and 26.0 mm, the differences in MedAEs were statistically significant in the ACD ≥ 3.5 mm subgroup (P = 0.018) (Table 4). In the ACD ≥ 3.5 mm subgroup, the MedAE predicted by the Haigis formula (0.29 D) was significantly smaller than other formulas except for the Holladay 1 formula (0.30 D) and MAE in the Haigis formula (0.33±0.37 D) was the lowest between four formulas. There was no correlation between the ACD and refractive errors predicted by the four formulas.

**Table 4:** Comparison of the median absolute error, mean absolute error and predicted refractive errors of subgroups based on the anterior chamber depth among the four formulas for intraocular lens power calculation in eyes with axial length 24.50–25.99 mm (Friedman test).

	Mean±SD				
	SRK/T	Holladay 1	Hoffer Q	Haigis	P-value
**2.5 mm ≤ ACD < 3.5 mm**					
**(n = 84)**					
**MedAE, D**	0.34	0.30	0.42	0.33	0.206
**MAE±SD, D**	0.45±0.41	0.43±0.40	0.56±0.45	0.41±0.34	
**RE±SD, D (range)**	0.02±0.58	-0.20±0.54	0.10±0.58	-0.10±0.53	
	(-1.97~1.37)	(-1.87~1.28)	(-1.98~1.41)	(-1.60~1.41)	
±0.25 D (%)	39.3	42.9	39.3	40.5	
±0.50 D (%)	64.3	70.2	63.1	67.8	
±1.00 D (%)	91.7	91.7	90.5	94.0	
>±2.00 D (%)	0	0	0	0	
**3.5 mm ≤ ACD**					
**(n = 79)**					
**MedAE, D**	0.33	0.30	0.39	0.29	0.018
**MAE±SD, D**	0.43±0.43	0.38±0.38	0.51±0.45	0.33±0.37	
**RE±SD, D (range)**	-0.01±0.61	-0.04±0.55	-0.04±0.65	-0.02±0.53	
	(-1.50~1.38)	(-1.39~1.49)	(-1.54~1.45)	(-1.49~1.57)	
±0.25 D (%)	39.2	44.3	37.2	49.4	
±0.50 D (%)	68.4	74.7	64.5	74.7	
±1.00 D (%)	92.4	94.9	91.1	97.5	
>±2.00 D (%)	0	0	0	0	

SD = standard deviation, ACD = anterior chamber depth, MedAE = median absolute error, MAE = mean absolute error, RE = mean predicted refractive error, D = diopters

In extremely long eyes with an AL more than 26.0 mm, the differences in MedAEs were also statistically significant in the ACD ≥ 3.5 mm subgroup (P = 0.001) (Table 5). At the ACD ≥ 3.5 mm, post hoc analysis showed that the Haigis formula and SRK/T formula were more accurate in calculating IOL power than the other two formulas (P < 0.0083), while there were no significant differences between the Haigis and SRK/T formulas (P = 0.136). Linear correlation analysis revealed no correlation between the ACD and refractive errors predicted by the four formulas in this subgroup.

**Table 5:** Comparison of the median absolute error, mean absolute error and predicted refractive errors of subgroups based on the anterior chamber depth among the four formulas for intraocular lens power calculation in eyes with axial length > 26.0 mm (Friedman test).

	Mean±SD				
	SRK/T	Holladay 1	Hoffer Q	Haigis	P-value
**2.5 mm ≤ ACD < 3.5 mm**					
**(n = 56)**					
**MedAE, D**	0.42	0.48	0.52	0.47	0.085
**MAE±SD, D**	0.56±0.58	0.62±0.55	0.68±0.55	0.53±0.45	
**RE±SD, D (range)**	-0.12±0.88	0.15±0.85	0.23±0.92	0.18±0.80	
	(-1.8~1.52)	(-1.64~1.93)	(-1.45~1.73)	(-1.39~1.43)	
±0.25 D (%)	32.1	26.8	25.0	30.3	
±0.50 D (%)	55.4	53.6	48.256	55.4	
±1.00 D (%)	83.9	85.7	80.4	89.3	
>±2.00 D (%)	0	0	0	0	
**3.5 mm ≤ ACD**					
**(n = 72)**					
**MedAE, D**	0.32	0.41	0.44	0.30	0.001
**MAE±SD, D**	0.46±0.54	0.54±0.52	0.60±0.54	0.43±0.55	
**RE±SD, D (range)**	-0.01±0.62	0.23±0.59	0.38±0.67	0.02±0.59	
	(-1.98~2.03)	(-1.58~2.12)	(-2.05~1.98)	(-2.12~1.99)	
±0.25 D (%)	38.9	30.6	27.8	44.4	
±0.50 D (%)	65.3	61.1	59.7	70.8	
±1.00 D (%)	87.5	86.1	84.7	91.7	
>±2.00 D (%)	1.4	1.4	2.8	1.4	

SD = standard deviation, ACD = anterior chamber depth, MedAE = median absolute error, MAE = mean absolute error, RE = mean predicted refractive error, D = diopters

## Discussion

The Hoffer Q formula is the best in short eyes,[3,4,11] with the SRK/T and Haigis formulas having better accuracy in long eyes.[3,12–15] All third-generation formulas document comparable accuracy in normal axial lengths as well as the Haigis formula.[3,4,8,17,19] The influence of axial lengths on refractive outcomes has been demonstrated. But, relatively few studies have reported the effect of different preoperative ACD among the IOL formulas in cataract patients. Jeong et al. reported that preoperative ACD has the greatest influence in IOL power calculation of the third-generation formulas and the Haigis formula.[20]

The present study evaluated the accuracy of the third-generation formulas and the Haigis formula according to ACD in eyes with short, normal, long and extremely long AL. Previous studies have reported the influence of ACD on short and normal eyes.[16,17] To our knowledge, no other studies explored the effect of ACD on long eyes. We followed the protocols introduced by Hoffer et al.[21] and obtained sufficient data to perform a statistical analysis.

In eyes with ALs < 22.0 mm, the Hoffer Q formula produced significantly more accurate ELP prediction than the Haigis formula in eyes with an ACD < 2.5 mm. This result is not consistent with the findings of Eom et al. that the Hoffer Q formula overestimates ELP in eyes with a short AL and a shallow ACD.[16] However, our data suggest that the Haigis formula underestimate ELP in eyes with a short AL and a shallow ACD. Muzyka-Wozniak and Ogar reported that the relative change in ACD after phacoemulsification measured with anterior segment optical coherence tomography (AS-OCT) was significantly larger in the eyes with AL < 22.0 mm than in the eyes with AL > 22.0 mm.[22] The Haigis formula uses a preoperative ACD to estimate ELP,[23] whereas the third-generation Hoffer-Q formula does not.[4] As ACD decreases, the Haigis formula might underestimate the relative change in ACD and could lead to the myopic predicted refractive error.

In normal eyes with AL of 22.0–24.5 mm, the accuracies of IOL formulas have been described.[3,4,8,19] Hoffer reported that the Hoffer Q and Holladay 1 formulas had better predictions than the SRK/T and Holladay 2 formulas in a study of 316 eyes.[8] Narvaez et al. examined 437 eyes and found no difference in the accuracy of the four IOL formulas (Hoffer Q, Holladay 1, Holladay 2 and SRK/T).[19] Aristodemou et al. studied 8,108 eyes and reported that the accuracy of the Hoffer Q, Holladay 1 and SRK/T formulas were comparable with AL 22.00–23.49 mm, while the Holladay 1 formula showed a slightly better accuracy than the Hoffer Q and SRK/T formulas in eyes with an AL of 23.50–24.49 mm.[3] However, these studies did not compare the effect of different ACD among the IOL formulas. Miraftab et al. reviewed 309 eyes with AL 22.00–24.49 mm and divided them into three subgroups (ACD ≤ 3.0, 3.0–3.5, and ≥ 3.5 mm). [17] The authors described that predictions with the Haigis formula were closest to emmetropia among the five formulas (Haigis, SRK-II, Hoffer Q, SRK/T and Holladay 1) at an ACD > 3.5 mm. In addition, the Haigis formula was significantly less accurate than the SRK/T and Holladay 1 formulas in the total sample. However, MAEs in each subgroup were not significantly different between the five formulas.[17] Presently, in eyes with AL 22.00–24.49 mm the Haigis formula might underestimate the ELP in eyes with an ACD < 2.5 mm and the relatively large number of samples with an ACD 2.50–3.49 mm reveals the superior accuracy of the Holladay 1 formula. This finding is consistent with previous studies.[3,8]

Previous studies recommended that the Holladay 1 formula as the most accurate in medium long eyes (24.5–26.0 mm).[3,8] In extremely long eyes (AL ≥ 26.0 mm), the SRK/T and the Haigis formulas are reportedly more accurate than other formulas.[3,12–15] Bang et al. reported that the Haigis formula performed better than the Holladay 1, Holladay 2, SRK/T and Hoffer Q formulas in all eyes longer than 27.0 mm.[12] Chen et al. also showed that the Haigis formula was the most accurate formula in myopic Chinese eyes with an AL > 26.0 mm.[13] Our results confirm that the Holladay 1 formula predicts ELP accurately in medium long eyes and the SRK/T formula predicts accurately in extremely long eyes. Also, the Haigis formula provided accurate ELP predictions with an ACD of ≥ 3.5 mm in eyes with an AL exceeding 24.5 mm. There are several explanations for this result. Several reports have documented that AL and ACD have no linear and positive correlation in long eyes.[24,25] Sedaghat et al. did not observe a linear relationship of AL and ACD in eyes with an AL exceeding 24.5 mm.[25] Chang and Lau also reported no correlation between AL and ACD in extremely long eyes (AL ≥ 27.5 mm).[24] This lack of correlation might influence the ELP predictions in third-generation formulas, which do not consider the preoperative ACD. The discrepancy between AL and ACD in long eyes is wider than that in the eyes < 24.5 mm, so the Haigis formula might show the better prediction than the Holladay 1 and Hoffer Q formulas in long eyes. Second, the Hoffer Q and Holladay 1 formulas limit superior ELP prediction to avoid overestimation of ELP in long eyes, while the Haigis and SRK/T formulas do not limit the superiority or inferiority of the ELP prediction. The Holladay 1 formula limits ELP prediction over eyes with an AL > 26.0 mm in a K-dependent fashion. The Hoffer Q formula has an upper limit of ELP of 6.5 mm.[26] Thus, these formulas might underestimate the ELP in long eyes with deeper ACDs.

The present study had some limitations. First, the sample size of small and there were relatively few long eyes. Second, we used bilateral eyes in the context of limited data especially in short and long eyes. However, we eliminated the variability between groups by using the Friedman non-parametric test, which is an appropriate statistical analysis. Lastly, we did not compare other promising fourth-generation formulas, such as the Holladay 2, Barrett and Olsen formulas, which consider more than two variables to predict ELP. Further large-scale studies including these formulas will be necessary.

In conclusion, the Hoffer Q formula performs best in short eyes with an ACD shallower than 2.5 mm. In short and normal eyes with an ACD < 2.5 mm, the Haigis formula might underestimate ELP and result in myopic predictive refractive error. On the other hand, the Haigis formula is recommended with its better prediction on ELP in eyes with an AL ≥ 24.5 mm and an ACD ≥ 3.5 mm.
